# Endovascular stent-graft procedures in aorto-iliac pathologies: a single center experience

**DOI:** 10.1186/1749-8090-8-S1-O111

**Published:** 2013-09-11

**Authors:** A Kucuker, M Hidiroglu, M Canyigit, L Cetin, F Saglam, E Sener

**Affiliations:** 1 Ataturk Training and Research Hospital, Cardiovascular Surgery Department, Karsiyaka-Izmir, Turkey; 2 Ataturk Training and Research Hospital, Radiology Department, Karsiyaka-Izmir, Turkey

## Background

We present our early results of endovascular treatments for the pathologies involving aorta and iliac arteries.

## Methods

117 endovascular stent-graft procedures in 113 patients for thoracic, abdominal and iliac pathologies was performed at our institute between January 2009-April 2013. 94(83.1%) patients were male. Indications for endovascular interventions were as follows; 58 for abdominal aortic aneursym, 14 for descending aortic aneursym, 4 for both descending and abdominal aortic aneursyms, 2 for thoraco-abdominal aneurysm including visceral arteries, 22 for aortic dissection, 2 for penetrating ulcer, 6 for traumatic aortic rupture, 1 for intramural hematoma, 1 for bronchial artery aneurysm and 3 for common iliac artery aneursym. Chimney and periscope techniques were used in 2 patients and iliac-branched endograft was placed in 2 patients.

## Results

25 of the 113 cases were treated emergently while the rest were elective procedures. Postoperative 30-day mortality was 6.19% (n=7). 5 of them had acute aortic pathologies (3 contained rupture,1 traumatic aortic rupture, 1 acute type-B dissection). The rest 2 patients died on the follow up period. Paraplegy occurred only in one patient(0.88%). Early endoleak rate was 21.2%, whereas late endoleak rate was 5.3%. 5 patients(4.2%) needed a second intervention. Visceral artery thrombosis was experienced in one patient during thoraco-abdominal aneurysm treatment with chimney technique,so the procedure was to be interrupted leaving abdominal aorta repair to a second stage.The overall technical success was 99.1%.Conversion to open surgery was not required in any patient.

## Conclusion

Endovascular aortic repair is an encouraging technique with low morbidity and mortality rates even in the emergency cases. Our early results of endovascular interventions correlate with the literature data.

